# Some Remarks Concerning Mercuric Bromide of Gold and Arsenic, with Especial Reference to Its Use in the Treatment of Neurotic Conditions of Specific Origin

**Published:** 1894-09

**Authors:** 


SOME REMARKS CONCERNING MERCURIC BROMIDE OF GOLD AND ARSENIC, WITH ESPECIAL REFERENCE TO ITS USE IN THE TREATMENT OF NEUROTIC CONDITIONS OF SPECIFIC ORIGIN.—At a recent meeting of this society, I had occasion to mention the use of the bromide of gold and arsenic in epilepsy. Being, comparatively speaking, a new drug to this market, it was thought of sufficient interest to bring it more prominently before the fellows.

The solution of bromide of gold and arsenic, after the formula of Dr. Barclay of Pittsburg, is of a beautiful red color, does not deposit, odorless, and tasteless; dosage ranging from five to twenty drops, each drop containing 1.32 of a grain of gold, 1.16 of a grain of arsenic, and 1.32 of a grain of mercury salts. It does not disagree with the stomach, nor relax the bowels. It will produce ptyalism, yet with less recession of the gums than is produced by mercury or the iodides.

Dr. Wood, in his admirable paper, read at the meeting of the Mississippi Valley Medical Association in Indianapolis, laid great stress upon its efficacy in diseases termed scleroses, incipient phthisis, arthritis deformans, in syphilitic diseases in its various forms, in hemiplegias, etc.

I have the pleasure of exhibiting three cases to-night, and a record of eight others, all of whom, with one exception, belong to the neuroses.

CASE 1.—No. 436 of my college clinical records; diagnosis hemiplegia. W. O. M., age twenty-eight, married; residence, Denver, Col.; family history, fairly good; occupation, proprietor of theater. Habits: had used alcoholic beverages rather freely for five years. February 28, 1893, following a two-days' drunk, patient vomited with great straining; passed into unconsciousness lasting seventy-two hours; complete dextra-hemiplegia, ataxia, aphasia sinistra-hemiplegia supervened. December 2, 1893, patient presented himself at clinic, having complete paralysis of the right side, could not count fingers with left eye; tongue on protrusion, pointing sharply to the right; corners of the mouth greatly depressed on right side; arm on the right side could scarcely be lifted away from the body; leg dragged after the patient in characteristic manner; all reflexes greatly exaggerated. Patient could answer yes and no to questions, but could not tell where he lived, and had to be brought to the college. Was placed on liq. arsenic et hydrarg. iodide, five drops in a wine glass of water, after meals.

December 6th, was given the mercuric bromide of gold and arsenic in five drops, doses after meals.

December 9th, patient developed la grippe, treatment was discontinued, and acetanilide given.

December 13th, condition improved, and mercuric bromide of gold and arsenic was given again.

December 20th, some improvement, medicine continued.

January 3, 1894, dose increased to ten drops.

January 26th, patient answers all questions with comparative ease, came to the clinic by himself, can lift his arm as high as his shoulder, the characteristic tremor taking place when he attempts to raise it still higher, can walk tolerably well; treatment continued.

February 7th, continued improvement.

February 14th, eating and sleeping as good as before attack.

February 28th, steady improvement in gait and speech.

March 10th, doing well.

March 17th, dose increased one drop every other day.

March 21st, has regained self-confidence.

April 4th, walks miles, does not have to use cane, no longer drags his foot. Secondary contraction of a slight degree has taken place in the fingers only. Students not familiar with the case could not detect any depression at an angle of the mouth. Sight in left eye much improved, though outer half is still impaired. Tongue points nearly in the median line. Patient steadily improving.

CASE 2.—J. R., aged thirty-five, tobacconist; personal and family history good; he has never had syphilis or rheumatism. His trouble began about eighteen months ago, with the characteristic pains in the lower extremities. These pains were felt at irregular intervals, sometimes daily and sometimes not for a space of a week or ten days. A short time afterward he noticed the change in gait. He also had frequent attacks of sick headache, three or four seminal emissions weekly, and difficulty in holding the urine. These symptoms ceased about four months ago, but the ataxia has greatly increased, and the pains still continue. He has noticed in the last three or four months a decline in the sexual power. He was admitted into the hospital February 1, 1894. The skin, reflexes, and the knee jerk were normal; there were no sensory symptoms; he had the Argyll-Robertson pupil; coördination was so imperfect that he was unable to stand with the eyes closed and the feet together; and he could hardly make his way from one place to another.

In treating this case I started February 28th, with eight drops of the mercuric bromide of gold and arsenic three times a day. From March 14th to April 1st, the dose was increased one drop each day. The total quantity given during thirty-three days being 1,245 drops, an average of thirteen drops at each dose.

CASE 3.—W. P., age thirty-two, painter; personal and family history good. His illness dates back to February 1, 1892, the symptoms being constipation, difficulty in voiding urine, stiffness and soreness in the knees. In a short time the legs became useless. In six weeks he was about on crutches, being at the time under treatment at Hot Springs. He entered the hospital July 7, 1892; at that time there was very little sensation in the left leg, the condition of the right being about normal. He had incontinence of urine and feces.

Commencing February 7th, I gave him five drops of the mercuric bromide of gold and arsenic after meals; the dose was increased daily until eleven drops were being given, making the average dose given during the course, of ten drops, thrice daily. Bladder and bowel symptoms restored.

CASE 4.—Mr. S. (I will refer to this case briefly, not being able to get the patient to come here to-night.) There was loss of sensation in right side of face; difficulty in coördination; ptosis; chronic constipation; loss of bladder control; reflexes lost. Four months' treatment followed by complete relief.

It could hardly be coincident that such decided changes for the better should have occurred in so short a time.

There have been and are now many preparations on the market which are not solutions of bromide of gold and arsenic, but bromide of gold with hydrobromic acid, arsenic acid, and arsenious acid. In the "Annual of the Universal Medical Sciences," issue 1891,

Dr. Hare, in his article on "Experimental Therapeutics," concludes in reefrence to the action of bromide of gold :

1. Bromide of gold undoubtedly inhibits the cortical motor centers, even when administered in smaller doses than other bromides. After an internal administration of bromide of gold in the dose of $1\frac{1}{2}$ to $3\frac{1}{10}$ grs. to the kilogramme of the animal's weight, even the strongest and very prolonged electric stimulation of the cortex fails to bring about any epileptic seizures. To obtain the same result from bromide of potassium, the latter should be introduced in the dose of $9\frac{1}{4}$ to $10\frac{3}{4}$ grs. to kilogramme. While bromide of sodium should be given in still larger quantities.

2. Irritability of individual motor centers, as determined by the appearance of contractions in corresponding muscular groups, is depressed by bromide of gold in but trifling degree.

3. Excitability of the white substance of the motor region remains intact.

4. The drug seems to affect mainly the tracts of communication between individual motor centers, as well as between remote areas of the cerebral cortex.

5. It does not appear to possess any special cumulative action. This action cannot be due to the amount of bromide present therein, since it is the weakest (55 per cent. by weight) of the three, while bromide of sodium is the heaviest (77.7 per cent.), the potassic salts standing midway.

In the "Annual of the Universal Medical Sciences," year 1892, an authority states " Better results can probably be obtained if the drug be given hypodermatically."

The opinion as to the efficacy of gold in disease has been indeed varied. In an article in Med. Rec. Vol. 34, page 802, September, 1888, this appears—referring to article on gold and arsenic : " It is highly digestible"; while farther on this startling statement appears : " There is probably not a sound clinical observation, showing that gold possesses any special therapeutic efficacy in any disease."

The old therapeutists limit its field of usefulness. Bartholow speaks most highly of it, recommending the double chloride in waning sexual force, in granular and fibrous kidney, in melancholia, in atonic dyspepsia, catarrh of bile ducts, etc. The claim made that it produces auric fever has not been observed, nor have I seen any evidences of gastric irritation. In a case of syphilitic nodules with marked cephalagra, there was a rapid subsidence of the nodes and relief of the head by the mercuric bromide of gold and arsenic, proving clearly that there is a most decidedly alterative influence exerted

The fact that the sedation produced by the bromide of gold is greater than that by the salts of potash and soda, should give it a front rank in the treatment of epilepsy, fortified by the alterative influence of the gold and the tonic effect of the arsenic. It has long been recognized that arsenic is the treatment for chorea. In three cases of this disease treated by bromide of gold and arsenic the results were all that could be desired.

The solution of gold is prepared by Johnson & Johnson.

1. Increase the appetite.

2. Do not irritate the stomach nor disturb the bowels, seeming to stimulate peristalsis without sufficient irritation to produce purgation.

3. Do exert decided powers of alteration, sedation where there is irritation, yet stimulating to waning powers.

4. Are indicated in the majority of diseases affecting coördination.

5. In fact, entitled to a trial in all neuroses.—THOMAS HUNT STUCKY, M.D., Professor of Theory and Practice of Medicine and Clinical Med., Hospital College of Medicine, Louisville, Ky., in *Medical and Surgical Reporter.*

